# Abundance and Distribution of Microplastics in Invertebrate and Fish Species and Sediment Samples along the German Wadden Sea Coastline

**DOI:** 10.3390/ani13101698

**Published:** 2023-05-20

**Authors:** Laura Polt, Larissa Motyl, Elke Kerstin Fischer

**Affiliations:** Microplastic Research at CEN (MRC, Center for Earth System Research and Sustainability), Universität Hamburg, Bundesstrasse 55, 20146 Hamburg, Germany

**Keywords:** *Arenicola marina*, *Littorina littorea*, *Mytilus edulis*, *Platichthys flesus*, biomonitoring, Nile red, mudflats

## Abstract

**Simple Summary:**

In order to reliably record pollution from microplastics within animals, indicators for a monitoring program must be found. For this purpose, we collected invertebrates, fish, and sediment cores at 10 sites along the Wadden Sea coast of Lower Saxony, Germany; determined the amount of microplastics; and made recommendations for potential bioindicator species, based on the sampling conditions and results. The species studied included lugworm (*Arenicola marina)*, common periwinkle (*Littorina littorea),* blue mussel (*Mytilus edulis),* and European flounder (*Platichthys flesus*). In total, microplastics were detected in 88% of the specimens and in each sediment core sample. Regarding the polymer composition, eight different types of microplastic were identified. Based on the results, the species blue mussel and European flounder are recommended for microplastic monitoring in biota.

**Abstract:**

Monitoring strategies are becoming increasingly important as microplastic contamination increases. To find potentially suitable organisms and sites for biota monitoring in the German Wadden Sea, we collected invertebrates (*n* = 1585), fish (*n* = 310), and sediment cores (*n* = 12) at 10 sites along the coast of Lower Saxony between 2018 and 2020. For sample processing of biota, the soft tissue was digested and the sediment samples additionally underwent a subsequent density separation step. Microplastic particles were identified using Nile red and fluorescence microscopy, followed by polymer composition analysis of a subset of particles via µRaman spectroscopy. All investigated species, sediment cores, and sites contained microplastics, predominantly in the morphology class of fragments. Microplastics were found in 92% of *Arenicola marina*, 94% of *Littorina littorea,* 85% of *Mytilus edulis*, and 79% of *Platichthys flesus*, ranging from 0 to 248.1 items/g. Sediment core samples contained MPs ranging from 0 to 8128 part/kg dry weight of sediment. In total, eight polymers were identified, predominantly consisting of polyethylene, polyvinylchloride, and polyethylene terephthalate. Considering the sampling, processing, and results, the species *Mytilus edulis* and *Platichthys flesus* are suitable species for future microplastic monitoring in biota.

## 1. Introduction

With the global increase in plastic and microplastic pollution, the establishment of monitoring strategies is currently the focus of national and international strategic developments [1,2,3,4]. Implementation of monitoring is primarily inhibited by the lack of uniform standard operating procedures for all environmental matrices. Furthermore, a successful implementation of monitoring programs must ensure the derivation of valid baseline and threshold values at a spatially and temporally representative resolution.

Microplastics (MPs) are commonly defined as solid synthetic polymer particles with a length of the maximum dimension below 5 mm [5,6,7] and are further subcategorized based on size, morphology, and chemical composition [8,9]. Marine environments are considered to act as a final sink for plastics [10,11], whereas terrestrial freshwaters act as transport pathways for plastics from land to the ocean, e.g., [12]. As for abiotic compartments, studies investigating the impacts of microplastics on organisms mainly focus on marine species [13], addressing the abundance and spatial distribution of MPs in species of different trophic levels and/or potential consequences associated with the uptake of microplastics and, consequently, the uptake of possibly toxic chemical compounds added or adsorbed to microplastics [14,15,16], as well as potential monitoring strategies [17,18,19].

Studies conducted in the greater North Sea and Baltic Sea area have considered fish species (23 publications found on google scholar for a total of 62 species from 2013 to 2022). Pelagic species such as *Clupea harengus* (Atlantic herring), *Trachurus trachurus* (Bastard mackerel), and *Sprattus sprattus* (European sprat) were considered in 13 of the selected publications on 34 species [20,21,22,23,24,25,26,27,28,29,30,31,32]. Moreover, 10 of the publications considered 27 demersal species such as *Melanogrammus aeglefinus* (haddock), *Pleuronectes platessa* (European plaice), and *Platichthys flesus* (European flounder) [23,24,25,27,29,30,31,32,33,34].

In terms of studies about MP abundance in invertebrates in the North and Baltic Sea, 17 publications on 18 species were considered. Despite the different and partly changing habitats of the investigated species, mainly filter feeders, especially *Bivalvia,* were included. Most of the available studies were on *Mytilus edulis* (blue mussel) (*n* = 15 publications, including [24,26,35,36,37,38,39]), followed by *Magallana gigas* (Pacific oyster) (*n* = 4, [24,37,39,40]). Gastropods were included in several studies. Here, the most studied species was *Littorina littorea* (common periwinkle), as the dominant grazer and suspension feeder (*n* = 4, [24,26,37,41]). Little studied in the North and Baltic Seas are the polychaetes, with two studies each addressing MP ingestion by *Arenicola marina* (lugworm) [24,39] and *Hediste diversicolor* (iridescent sea annelid) [42,43].

Sediment studies of the North Sea mudflats are very limited, and the MP concentrations vary significantly between studies. In particular, the heterogeneity of the applied methods of sample preparation and MP identification, and the heterogeneity of the lower detection sizes between 1.2 µm and 500 µm, have to be considered. In addition, different reference values, such as concentrations per weight or volume, and different reporting of concentrations in terms of ranges, means, or medians were used. All these factors largely prevent comparison of the results.

The monitoring of MP in biota is currently receiving increased attention on behalf of the Marine Framework Strategy Directive [44] and regional sea conventions, such as the Convention for the Protection of the Marine Environment of the North-East Atlantic (OSPAR) [45] and the Helsinki Commission (HELCOM) [46]. Among the potential species for MP monitoring in biota, mussels are the most common that are currently taken into consideration [19,47,48,49]. However, in terms of the identification and evaluation of MP monitoring species, only a few studies have considered multiple phyla and species. This poses a problem, since a reasonable monitoring strategy should take into account a selection of suitable indicator organisms, as representative as possible of the regions of interest. Another problem in the planning of suitable monitoring strategies is the lack of comparable data. Only if valid data sets are available is it possible to decide on the localization of monitoring sites and the required monitoring frequencies.

In the present study, we analyzed microplastics in biota along the German North Sea coast, as a first approach to a possible future monitoring strategy in the area.

The objectives of the current study were accordingly:

1. Analyzing the nature and extent of the occurrence of microplastics in biota in the North Sea coastal waters of Lower Saxony.
2. Evaluation of species–specific differences and possible correlations with species- and individual-specific parameters.
3. Evaluation of spatial differences and influencing factors of the occurring microplastic concentrations in biota between selected study stations along the coast of Lower Saxony.
4. Provision of recommendations for a future monitoring strategy on microplastics in biota for the Lower Saxony coastal waters, with regard to the selection of indicator organisms, station selection, monitoring frequency, and analysis methodology.

## 2. Materials and Methods

### 2.1. Study Area and Sampling

Invertebrate and fish species were investigated within a project on microplastic abundance and distribution in biota of the Wadden Sea coastline of Lower Saxony, Germany on behalf of the Lower Saxony State Agency for Water Management, Coastal Defence, and Nature Conservation (NLWKN, Oldenburg, Germany). The sample areas are located within the Wadden Sea National Park, which stretches from the Dutch border westsides to the river Elbe eastsides, covering a total area of 3450 square kilometers [50]. The flat coastal region of the Wadden Sea consists of large areas of tidal flats that regularly dry out [51]. The climate is characterized by a warm and humid temperate climate, with a mean annual temperature of 9.7 °C and an annual precipitation of 752 mm (station Norderney, [52]).

**Figure 1:**
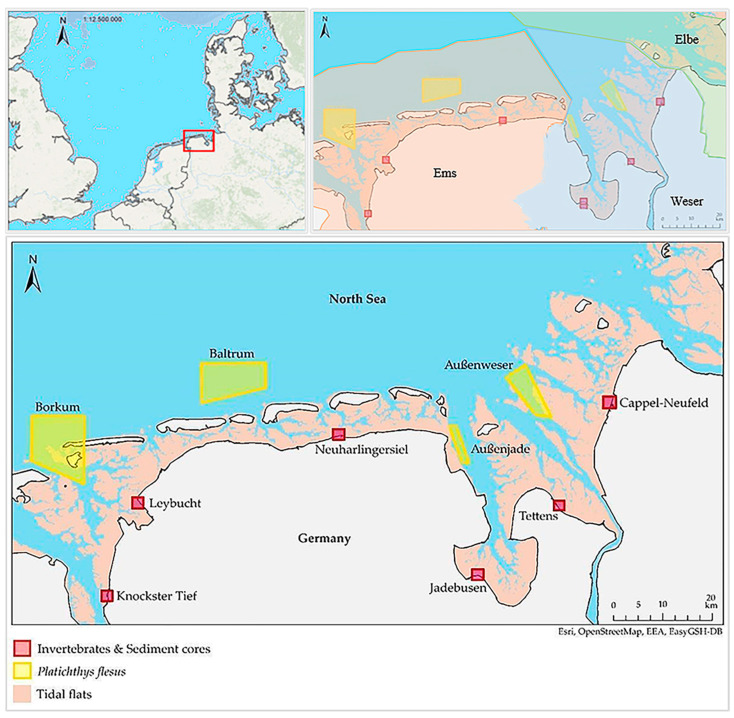
Sample area in Northern Germany (**top left**); location of river estuaries (**top right**); and sampling locations of invertebrate species, sediment cores, and *Platichthys flesus* (**bottom**) along the German Wadden Sea coastline of Lower Saxony, with representation of the mean tidal level.

Invertebrates and sediment cores were sampled in autumn 2019 and summer 2020, while fish were sampled in the summers of 2018 and 2020. The locations were selected corresponding to the sampling stations of campaigns within the Federal and State Measurement Programme on contaminants (Figure 1). The stations differ in exposure and location in relation to water/tidal currents, as well as potential microplastic sources, tidal flat types, and anthropogenic pressure (Table 1).

Invertebrates: A total number of 1585 individuals were taken at 6 sampling stations, comprising *Arenicola marina* (*n* = 308, pooled by ~5 individuals resulting in 62 pooled groups), *Littorina littorea* (*n* = 583, pooled by ~15 individuals—39 pooled groups), and *Mytilus edulis* (*n* = 694, pooled by ~5 individuals—139 pooled groups). The pooling of organisms was performed in order to receive a microplastic signal well above the detection limit. The number of individuals per pooled group was determined based on the experience of the comparative study on the Schleswig-Holstein Wadden Sea coast [24]. The individuals that were pooled together showed similar characteristics with respect to their size and were all taken at the same sampling spot.

The species were selected according to discussions with the Lower State Agency for Coastal Protection, meeting the criteria of being species with predominant abundance throughout the regions and representing different feeding types. *Arenicola marina* (*polychaeta*) represents an almost ubiquitous deposit feeder in the Wadden Sea, preferring muddy to sandy substrates and mainly staying at about 30 cm depths. *Littorina littorea* (*gastropoda*) is a common grazing feeder in the region that can be found on stony surfaces or on sandy to muddy substrates closer to the shoreline of the mudflats. *Mytilus edulis* (*bivalvia*) is the predominant filter feeder, with frequent occurrence in stony shoreline sections and in mussel banks, but it also occurs on the surface of the preferred muddy to sandy substrates. Depending on the different habitats, not all species could be sampled at all stations (see Table S1a Supplementary Material).

Invertebrate samples were taken manually, rinsed with filtered MilliQ-water, and immediately frozen at −18 °C.

Fish: A total of 310 individuals of *Platichthys flesus* were collected from four fishing areas. Fish species were caught during regular fish monitoring cruises carried out by Nowak GmbH on behalf of the Lower Saxony State Agency for Water Management, Coastal Defence, and Nature Conservation. Fish individuals were placed into polyethylene bags and frozen at −8 to −18 °C and transported in freezer boxes for further processing in the laboratory.

Sediment: Sediment samples were retrieved using polyvinyl chloride (PVC)-corers with a diameter of 5 cm, down to the maximum feasible depth of about 30 cm.

### 2.2. Laboratory Analysis—Sample Treatment

Invertebrate: Every individual was photographed, and their length, width, net weight (weight of dissected soft tissue), and gross weight (weight of the total individual including shell if present) were recorded. For the extraction of microplastics from individuals, the soft tissue was removed from the shell for *Mytilus edulis* and *Littorina littorea*. The weight of the tissue per individual was determined with an analytical balance to an accuracy of 0.01 mg and transferred to previously pooled entities. *Arenicola marina* was transferred as whole individuals into beakers.

Fish: For the analysis of *Platichthys flesus*, only the gastrointestinal tract was taken into account. Tissues were weighed and transferred into precleaned glass beakers, each containing the tissue of *n* = 1 individual.

For digestion of biogenic organic matter, 10 mL of a solution consisting of potassium hydroxide (KOH) and sodium hypochlorite (NaClO) per gram of wet weighed tissue was added. The digestion solution was prepared according to Strand and Tairova, using a mixture of 150 mL NaClO (6–14%), 300 mL KOH (10 M), and 550 mL MilliQ-water [53], pre-filtered <1.2 µm to minimize contamination. Samples were incubated for 48 h at room temperature, and in case of visually detectable incomplete digestion, the samples were additionally heated to 40 °C and agitated at low speed. The digested sample suspensions were passed over a stainless steel analytical sieve with a mesh size of 20 µm, rinsed with MilliQ-water, and filtered in a stainless steel filtration unit (Sartorius Combisart) with paper filters (VWR, qualitative filter paper 413, 5–13 µm particle retention). Finally, filters were transferred for drying into acetone-rinsed glass petri dishes.

Sediment: For microplastic analysis the sediment cores were cut into 5 cm sections. In total, 56 subsamples were generated (3 to 6 sediment horizons per sediment core, depending on the total depth). To avoid contamination from the PVC tube, the outermost part of the sample was not used for microplastic analysis. The samples were homogenized, and 50 mL was measured and weighed into a glass beaker [53]. Digestion of biogenic organic matter was performed via a modified protocol according to Hengstmann et al. [54]. Then, 100 mL of hydrogen peroxide (H_2_O_2_, 10%) was added and left to react for 7 days at room temperature. Following a rinsing step over a 20 µm sieve, a second digestion step with the addition of 50 mL NaClO (6–14%; volume ratio 1:3) and a reaction time of 48 h at 40 °C was included. Subsequently, a density separation with sodium iodide (NaI, 1.6 g cm^−3^) was undertaken in a specific glass separation column. The sample/NaI suspension was shaken overhead 12 times and left to settle for 15 min. The settled sediment was carefully extracted through the outlet at the bottom of the column. The remaining supernatant was filtered onto a paper filter (VWR, qualitative filter paper 413, 5–13 µm particle retention) with a stainless steel filtration device (Sartorius Combisart) and placed into acetone-rinsed petri dishes.

Blank samples were processed throughout the analyses according to the sample series, and the resulting values were subtracted from the sample results.

### 2.3. Identification of Particle Characteristics and Polymer Composition

For identification, the Nile red staining method in combination with fluorescence microscopy (AxioLab A.1, Carl Zeiss Jena, TRITC HC Filterset (AHF), 2.5×) was applied [55,56]. Particle dimensions of potential MP particles were recorded, and particle morphology was determined. A random subset of particles (*n* = 208) was investigated for polymer composition using µRaman spectroscopy (DXR2xi Raman Imaging Microscope, Thermo Fisher Scientific, Waltham, MA, USA).

### 2.4. Statistical Analysis

The data resulting from the invertebrate pooled groups were recalculated to the number of individuals per group (items/individual) and the total weight of tissue of all grouped individuals (items/g). Statistical analyses were performed using R statistics (R Core Team 2021, Vienna, Austria, version 4.1.2) in an R Studio environment (RStudio Team 2021, Posit PBC, Boston, MA, USA version 2021.09.1) and IBM SPSS Statistics (IBM Corp. 2019, version 26.0, Arbank, NY, USA). A Shapiro–Wilk test was applied to test the normal distribution of microplastic concentrations. Since the data were non-parametric, median values are given throughout the manuscript (all descriptive parameters are provided within the Supporting Information). Depending on the distribution of the parameters, tests for differences in means were performed using a Kruskal–Wallis test followed by Bonferroni correction, and correlation analyses of microplastic concentrations with individual-specific parameters were calculated according to SPEARMAN. The significance level was set at α = 0.05 (α = 0.01 when indicated). Results were visualized using the R library ggplot2 [57].

### 2.5. Background Contamination

Precautions were taken to minimize background contamination. Therefore, all chemical solutions and MilliQ-water were filtered (691, VWR International, 1.6 μm retention), glass materials were used and pre-rinsed with acetone and MilliQ-water. Beakers were covered during standing hours, cotton laboratory coats were worn at every processing step, as well as during the integration of procedural blanks (*n* = 94). The humidity was increased and air filtration devices were used in the laboratory [58,59].

## 3. Results

### 3.1. Procedural Blanks

An average contamination of 3.9 particles (comprising 3.7 fragments and 0.2 fibers) was found in the procedural blank samples investigated alongside the invertebrate samples (*n* = 82). Blank samples were assigned to the analyzed composite sample and subtracted accordingly. Concerning the sediment procedural blanks (*n* = 12), a total number of six fragments and six fibers were found. The mean MP value of the blanks was subtracted from the results of the sediment samples; resulting in the subtraction of 2.5 fragments and 3.2 fibers per sample.

### 3.2. Invertebrates and Fish

#### 3.2.1. Microplastic Distribution and Concentrations

A large proportion of individuals from all species were affected by MP contamination (88%). The highest ratio was determined for *Littorina littorea* (94%), whereas lowest number of affected individuals was determined for *Platichthys flesus* (79%, Table 2).

The MP concentrations detected in invertebrate species varied considerably (Table S1a–c descriptive statistics according to items/g and particle morphology). In the following, median concentrations are given in particles per gram of analyzed tissue (items/g). Please refer to Table S2a–c for particle concentrations per individual. All levels of significance were set to α = 0.05.

Considering all species investigated, the concentrations ranged from 0 to 248 particles per g of analyzed tissues (given as items/g in the following). Amongst species, the concentrations followed the order from the lowest concentrations in *Platichthys flesus* (median over all samples 0.5 items/g), *Littorina littorea* (median 2.5 items/g), *Mytilus edulis* (median 3.0 items/g), and the highest concentrations in *Arenicola marina* (median 4.7 items/g).

**Figure 2:**
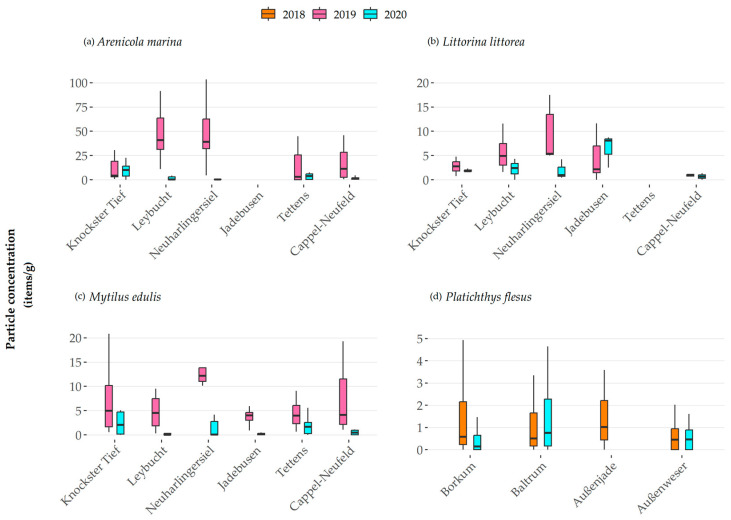
Microplastic concentrations (n per g weight of analyzed tissue) by invertebrate species and *Platichthys flesus* gastrointestinal tract per year and sampling location. (Outliers are excluded, please note different y-scale dimensions). No individuals could be sampled of *Arenicola marina* at Jadebusen, *Littorina littorea* at Tettens in 2019 and 2020, nor *Platichthys flesus* at Außenjade in 2020. This was due to habitat preferences of *Arenicola marina,* with the location Jadebusen being more sandy than the other locations. *Littorina littorea* does not occur along this shoreline section at all. *Platichthys flesus* could not be sampled in one season, due to the weather conditions.

Regarding the sampling stations and considering the different measuring campaigns, the highest MP concentrations in *Arenicola marina* (Figure 2a) were detected at Leybucht 2019 (median 40.8 items/g), Neuharlingersiel 2019 (median 39.2 items/g), and Cappel-Neufeld 2019 (median 11.4 items/g); followed by Knockster Tief 2020 (median 10.0 items/g), Knockster Tief 2019 (median 4.3 items/g), Tettens 2020 (median 3.9 items/g), Tettens 2019 (median 3.1 items/g), and Cappel-Neufeld 2020 (median 1.1 items/g); with the lowest at Neuharlingersiel 2020 (median 0.4 items/g) and Leybucht 2020 (median 0.4 items/g). Regardless, no significant differences were detected. In *Littorina littorea* (Figure 2b), the highest amounts of MP particles per g analyzed tissue were similarly found in Jadebusen 2020 (median 8.1 items/g), Neuharlingersiel 2019 (median 5.4 items/g), and Leybucht 2019 (median 4.9 items/g). Lower concentrations were found in Knockster Tief 2019 (median 2.8 items/g), Leybucht 2020 (median 2.4 items/g), Jadebusen 2019 (2.2 items/g), and Knockster Tief 2020 (median 1.8 items/g). Lowest concentrations were found at Cappel-Neufeld 2019 (median 1.9 items/g), Neuharlingersiel 2020 (median 1.0 items/g), and Cappel-Neufeld 2020 (median 0.7 items/g). Considering all MP particles, regardless of their morphology, a significant difference in concentrations could be observed between individuals from Neuharlingersiel and Cappel-Neufeld (*p* = 0.025). *Mytilus edulis* (Figure 2c) specimens showed the highest concentrations at Neuharlingersiel 2019 (median 12.2 items/g), Knockster Tief 2019 (median 5.0 items/g), and Leybucht 2019 (median 4.5 items/g), followed by Cappel-Neufeld 2019 (median 4.1 items/g), Jadebusen 2019 (median 4.0 items/g), and Tettens 2019 (median 3.9 items/g). The stations with the lowest concentrations were Knockster Tief 2020 (median 2.1 items/g), Tettens 2020 (median 1.7 items/g), Cappel-Neufeld 2020 (median 0.5 items/g), Jadebusen 2020 (median 0.2 items/g), Neuharlingersiel 2020 (median 0.1 items/g), and Leybucht 2020 (median 0.0 items/g). Significant differences in fiber concentrations were detected between Neuharlingersiel and the stations Knockster Tief (*p* = 0.028) and Jadebusen (*p* < 0.001), as well as between Jadebusen and the stations Leybucht (*p* = 0.002) and Tettens (*p* = 0.015). The fish species *Platichthys flesus* (Figure 2d) showed the highest contamination at the station Außenjade 2018 (median 1.0 items/g), followed by Baltrum 2020 (median 0.5 items/g), Borkum 2018 (median 0.6 items/g), Baltrum 2018 (median 0.5 items/g), Außenweser 2020 (median 0.5 items/g), Außenweser 2018 (median 0.4 items/g), and Borkum 2020 (median 0.1 items/g). Differences in concentrations were significant between the station Jadebusen and both Außenweser (*p* = 0.002) and Borkum (*p* = 0.002).

MP concentrations varied according to the year and season of the sampling campaigns. In the case of *Arenicola marina* individuals, significant differences between the sampling campaigns at Leybucht (2019 median 40.8 items/g, 2020 median 0.4 items/g, *p* < 0.001), Neuharlingersiel (2019 median 39.2 items/g, 2020 median 0.4 items/g, *p* < 0.001), and Cappel-Neufeld (2019 median 11.4 items/g, 2020 median 1.1 items/g, *p* = 0.007) were detected. *Littorina littorea* samples showed a significant difference at Neuharlingersiel (2019 median 5.4 items/g, 2020 median 1.0 items/g, *p* = 0.009). Across all sampling stations, the MP concentrations were significantly different for *Mytilus edulis *(*p* ≤ 0.001–0.033). Regarding *Platichthys flesus*, a significant difference was seen at Borkum (2018 median 2.4 items/g, 2020 median 0.66, *p* < 0.001).

Correlations with regards to the MP concentrations per g net weight and basic data of the specimen could be detected. For *Arenicola marina*, a significant positive correlation between the amount of fibers and individual net weight (r = 0.518, α = 0.01), length (r = 0.484, α = 0.01), and width (r = 0.344, α = 0.01), as well as between the amount of fragments and individual net weight (r = 0.211, α = 0.01), length (r = 0.192, α = 0.01), and width (r = 0.145, α = 0.05) was found. For *Mytilus edulis*, significant positive correlations were found between fibers and individual net weight (r = 0.386, α = 0.01), length (r = 0.464, α = 0.01), and width (r = 0.428, α = 0.01), as well as between fragments and individual length (r = 0.133, α = 0.05). A positive correlation was also observed for *Platichthys flesus* between fragments and net weight (r = 0.120, α = 0.01). No significant correlations were detected for *Littorina littorea.*

#### 3.2.2. Particle Characteristics

The ingested particles predominantly consisted of fragments (92%), followed by fibers (5%) and microbeads (3%) (Table 3). The overall size distribution of fragments reveals that 44% were smaller than 50 µm, with progressively increasing frequencies with decreasing particle sizes. Fragments displayed a median length of 55 µm and ranged from 20 µm to 1907 µm. The fibers showed no particular size distribution, ranging from 45 µm to 4990 µm (median 573 µm). Microbeads were found in all species and were smaller than 1000 µm, with a median length of 37 µm.

Concerning the particles found in *Arenicola marina*, the smallest fragment sizes at the stations were identified at Leybucht (median 47 µm), followed by Knockster Tief (median 52 µm), Neuharlingersiel (median 54 µm), Cappel-Neufeld (median 57 µm), Tettens (median 57 µm), and Jadebusen (median 64 µm). Comparing the sampling campaigns, significant differences in particle lengths only occurred at the station Cappel-Neufeld (*p* < 0.001, α = 0.05). No significant differences between sampling stations for fiber length could be determined; however, the size of fragments differed significantly between the stations Leybucht and Jadebusen (*p* = 0.013, α = 0.05), Knockster Tief and Cappel-Neufeld (*p* = 0.026, α = 0.05) and Jadebusen (*p* < 0.001, α = 0.05), Neuharlingersiel and Cappel-Neufeld (*p* = 0.025, α = 0.05) and Jadebusen (*p* < 0.001, α = 0.05), and Tettens and Jadebusen (*p* = 0.018, α = 0.05). The median lengths of fragments found in *Littorina littorea* were smallest at Leybucht (median 47 µm), followed by Jadebusen (median 48 µm), Knockster Tief (median 54 µm), Cappel-Neufeld (median 55 µm), and Neuharlingersiel (median 59 µm). However, no significant differences in fibers nor in fragments according to the different sampling stations and sampling campaigns were found. Regarding the fibers found at *Mytilus edulis*, no significant differences in size between sampling locations were detected. Considering the fragments, the median particle size were smallest at Cappel-Neufeld (48 µm), followed by Neuharlingersiel (53 µm), Knockster Tief (55 µm), Tettens (57 µm), Jadebusen (62 µm), and Leybucht (81 µm). The differences in size were significant between sampling locations (*p* ≤ 0.001–0.003, α = 0.05), except for Neuharlingersiel vs. Knockster Tief and Tettens, Knockster Tief vs. Tettens, and Jadebusen vs. Tettens and Leybucht. The smallest fragments in *Platichthys flesus* were recorded at Baltrum (median 51 µm), followed by Außenjade (median 56 µm), Außenweser (median 67 µm), and Borkum (median 68 µm). All particle sizes differed significantly (*p* < 0.001, α = 0.05) between sample locations, except for Baltrum vs. Jadebusen and Borkum vs. Außenweser.

**Figure 3:**
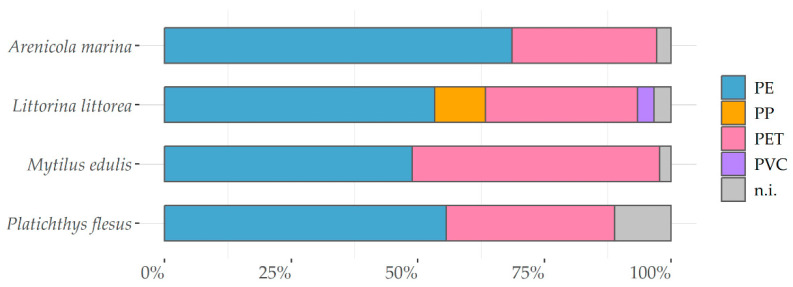
Percentage distribution of polymer types based on identification with μRaman spectroscopy per invertebrate species and *Platichthys flesus*. (*Arenicola marina* (*n* = 35), *Littorina littorea* (*n* = 32), *Mytilus edulis* (*n* = 46), and *Platichthys flesus* (*n* = 27).

A random subsample of 140 particles were analyzed for polymer composition using µRaman spectroscopy. Of these, 134 particles were verified as synthetic polymers with four different polymer types. The predominant type found in the samples was PE (59%), followed by PET (38%). One PP and one PVC particle were also found in *Littorina littorea.* The distribution per species is shown in Figure 3. In consideration of the particle morphology, the analyzed fibers (*n* = 47) consisted exclusively of PET.

### 3.3. Sediment Cores

#### 3.3.1. Microplastic Distribution and Concentrations

The sediment samples across all stations and depths contained MPs ranging from 0 to 8128 particles per kg dry sediment (median 2775 part/kg DS).

**Figure 4:**
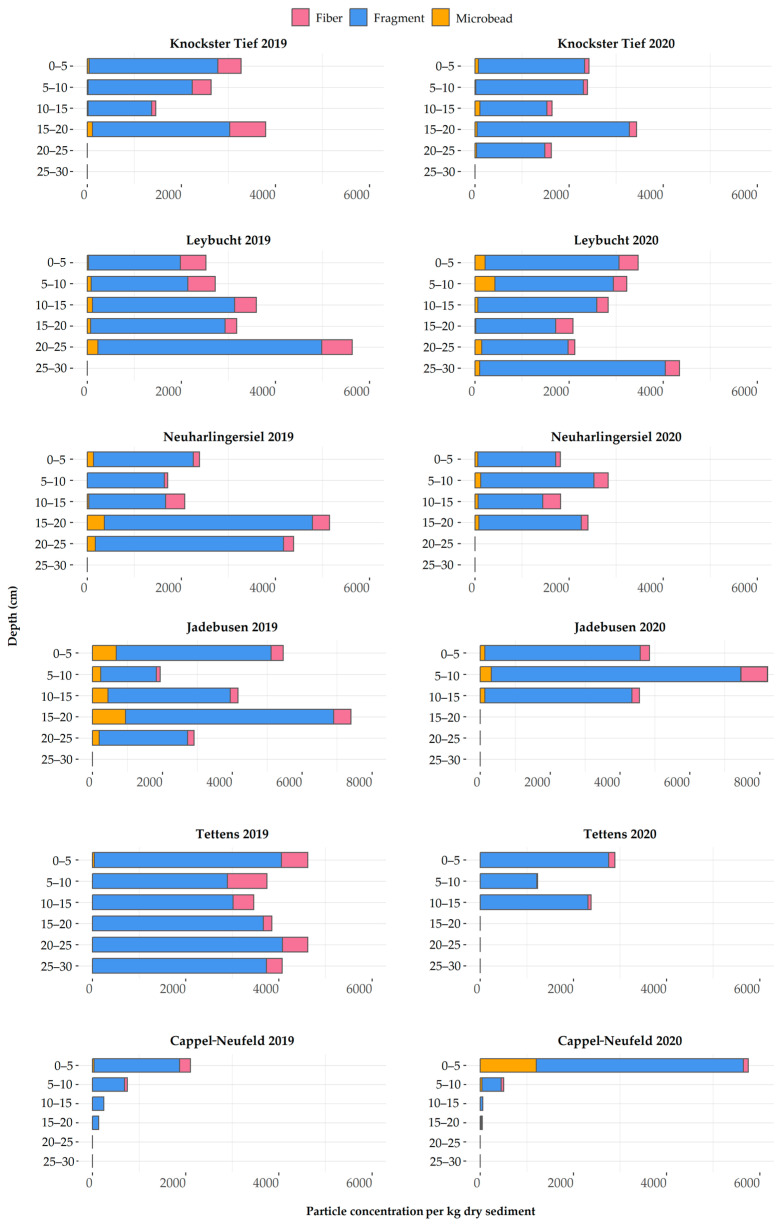
Microplastic concentrations (per kg dry sediment) divided by morphology and their distribution within the different sediment depth profiles for the years 2019 and 2020.

Considering the findings per station and sampling year (Figure 4), Tettens 2019 showed the highest MP concentration (24,362 part/kg DS), followed by Jadebusen 2019 (21,855 part/kg DS), Leybucht 2020 (18,073 part/kg DS), Leybucht 2019 (17,643 part/kg DS), Jadebusen 2020 (17,527 part/kg DS), Neuharlingersiel 2019 (15,705 part/kg DS), Knockster Tief 2020 (11,510 part/kg DS), Knockster Tief 2019 (11,154 part/kg DS), Neuharlingersiel 2020 (8868 part/kg DS), Tettens 2020 (6500 part/kg DS), Cappel-Neufeld 2020 (6348 part/kg DS), and Cappel-Neufeld 2019 (3224 part/kg DS) (Table S3: descriptive statistics of MP concentrations in sediment according to locations and depths). Regarding the depth profiles, no MP distribution pattern could be detected.

#### 3.3.2. Particle Characteristics

Fragments represented the dominant morphology class, with 86%, followed by fibers (10%) and microbeads (4%). The size of fragments varied between 20 µm and 1383 µm, with a median length of 46 µm. The particle amount increased with decreasing particle size, resulting in 56% of all particles being smaller than 50 µm. Fibers ranged from 58 µm to 4706 µm (median 749 µm), without a particular size distribution. Regarding the microbeads, the sizes were smaller than 400 µm, with a median size of 44 µm, and were found across all stations within different depths, with the exception of the 2020 Tettens core, which did not contain microbeads in any layer.

Concerning the particle sizes, the smallest fragment sizes at the stations were identified at Leybucht (median 47 µm), followed by Knockster Tief (median 52 µm), Neuharlingersiel (median 54 µm), Cappel-Neufeld (median 57 µm), Tettens (median 57 µm), and Jadebusen (median 64 µm).

The polymer composition was analyzed for a random subsample of 68 particles using µRaman spectroscopy. Polyvinyl chloride (PVC 68%) was the dominant polymer type, followed by polyethylene terephthalate (PET 14%), polystyrene (PS 6%), poly(methyl methacrylate) (PMMA 3%) and polyethylene (PE 2%), polypropylene (PP 2%), polyamide (PA 2%), and polyoxymethylene (POM 2%). The fibers analyzed (*n* = 6) consisted exclusively of PET, whereas the microbeads analyzed (*n* = 6) were made of PVC (*n* = 3) and PS (*n* = 3).

**Figure 5:**
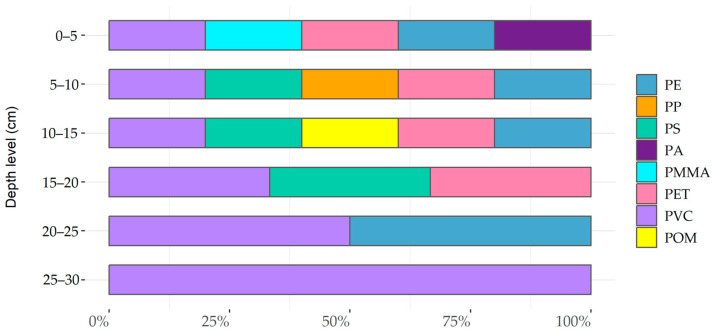
Percentage distribution of polymer types (*n* = 68) based on identification with μRaman-spectroscopy of sediment cores per depth level (cm). In total, *n* = 20 were identified for the depth 0–5 cm, *n* = 12 for 5–10 cm, *n* = 12 for 10–15 cm, *n* = 12 for 15–20 cm, *n* = 6 for 20–25 cm, and *n* = 1 for 25–30 cm.

Regarding the distribution of depth profiles (Figure 5), it is noticeable that PVC particles were found at every depth level.

## 4. Discussion

### 4.1. MP Concentrations in Invertebrates and Fish

The percentage of affected individuals revealed that MP contamination is ubiquitous. However, the MP particle concentrations varied according to the investigated species, locations, and seasons.

In general, the highest concentrations detected for the species *Arenicola marina* were very pronounced for the sampling season in early autumn 2019 at the stations Leybucht and Neuharlingersiel. The station Leybucht is characterized by its location in the wider estuary of the Ems river, behind the barrier isles of Borkum, Juist, and Norderney, and therefore represents an area of elevated sediment deposition rates. Neuharlingersiel is also located behind barrier isles (Langeoog and Spiekeroog); however, it is not affected by potential MP inputs via a river or estuary. Neuharlingersiel represents a station with high tourist frequentation and is one of the central ferry ports on the German North Sea coastline. The exposure to MP sources and the specific sediment deposition conditions here are likely to play an important role concerning the ingestion of MPs through the deposit feeder Arenicola. However, the MP concentrations at both stations during the sampling season summer 2020 were within the same range as at the other sampling stations, and no significant differences were detected. Taking a closer look at the dependencies of MP concentrations and individual characteristics, a negative correlation between MP concentrations and individual weight, length, and width was detected (correlation coefficients −0.70, −0.54, and −0.49, α = 0.01); whereby, the smaller the individual, the higher the contamination. This contradicts the hypothesis that MPs in *Arenicola marina* only reflect a distinct sediment background signal and is supported by the finding that the *Arenicola marina* individuals taken at the stations Leybucht and Neuharlingersiel in autumn 2019 were significantly smaller than those sampled in summer 2020. This was most likely due to seasonal movements of *Arenicola marina* from the mid-tidal levels to upper shore regions, because of the migration of juveniles [60], which should be considered in future studies, to avoid or to further investigate this bias. As shown in the mesocosm experiments, *Arenicola marina* practices size-selective feeding, also leading to the accumulation of MPs in the feeding layer between 10 and 15 cm sediment depth [61]. Such an accumulation depth could not be verified within the sediment cores; however, it has to be considered that other bioturbation species and highly specific deposition mechanisms are present in the mudflats.

In general, our findings for MP concentrations in *Arenicola marina* are higher than those found in the study of Van Cauberghe et al., who found 1.2 ± 2.8 items/g (lower size cutoff 5 μm) in *Arenicola marina* on the French–Belgian–Dutch coastline [39]. A previous study from the North Sea of Schleswig Holstein showed a concentration of 3.6 items/g (mean) in *Arenicola marina*, which is also higher than the results found in the present study [24]. These differences are predominately related to methodological differences, especially in terms of the lower cut-off size. Compared to the study carried out in the German Wadden Sea along the coastline of Schleswig-Holstein [24], the concentrations for the southern Wadden Sea coastline investigated in this study are almost twice as high; however, when comparing using a targeted lower size cut-off recalculated to 63 µm, the results are in line with the median concentration of 2.3 items/g in Schleswig-Holstein and 2.1 items/g in Lower Saxony.

In terms of the particle characteristics and the significant differences in fragment lengths identified in *Arenicola marina* between the sampling stations, the conditions of different MP exposures from potential sources, and above all the sedimentation conditions and the underlying basin region expositions, morphologies, and currents during tides are most likely responsible. This is underlined by particles that were significantly larger in the eastern sampling stations of the Weser estuary compared to the more western stations, which additionally all are located behind the barrier isles along the German southern North Sea coastline. The subset of MP particles from biota samples analyzed for their polymer composition were mainly made of lightweight PE fragments, whereas the fibers were made of PET. This finding applies for all samples derived from biota samples; however, it has to be considered that the total amount of particles analyzed was low, due to a lack of resources, and thus cannot be considered fully representative.

MP concentrations detected in *Littorina littorea* individuals were lower compared to *Arenicola marina* and in line with the concentrations detected for *Mytilus edulis*. As shown for *Arenicola marina*, the MP concentrations at the stations Leybucht and Neuharlingersiel and as well Jadebusen (not investigated for *Arenicola marina*) exhibited the largest concentrations and differences according to the sampling campaigns. This increases the evidence of the stated influence of station characteristics, such as exposure to potential MP sources and specific conditions of hydrodynamics and sedimentation. No correlation between MP concentrations and individual characteristics of size and weight was present. Comparable data for *Littorina littorea* have been found on the west coast of Ireland, with highest concentration of microplastics at the location of Blackhead (2.96 ± 2.92 items/g, lower size cutoff 1.2 μm) [41]. This value is comparable with the station Knockster Tief (median 2.8 items/g), one of the locations in this study exhibiting lower concentrations. A much higher concentration was found in the Dutch river delta, with a total of 20 items/g (lower size cutoff 0.7 μm) for 10 pooled individuals [37], as well as along the Schleswig-Holstein coastline in Germany [24], with a mean of 15.90 items/g (lower size cutoff 63 μm). The latter study revealed concentrations several magnitudes higher compared to this study, accounting for 5.5 items/g in Schleswig-Holstein, especially when recalculated to the respective size range (>63 µm), which results in an MP concentration of 0.9 items/g in Lower Saxony.

Differences in particle characteristics regarding *Littorina littorea* in terms of length of morphologies were not significant. In comparison to *Arenicola*, no size-selective feeding behavior was demonstrated for the grazer Littorina. The polymer composition of MP particles found in *Littorina littorea* were similar to the findings for the other investigated species, as described for *Arenicola marina*.

The same pattern across sampling stations and campaigns as for *Arenicola marina* and *Littorina littorea* was also shown for *Mytilus edulis*; however, higher concentrations were also present at the stations Knockster Tief and Cappel-Neufeld. The higher MP concentrations in samples from the sampling campaign carried out in autumn 2019 compared to summer 2020 (*p* < 0.001) points to the importance of defining the right time and season for Mytilus sampling. It is known that environmental factors, such as algal concentration and acidification, influence the filtration performance of *Mytilus edulis*, which probably also played a role here [62]. Data collected from different seasons (January, May, and July) also demonstrated that the clearance rate of *Mytilus edulis* was independent of seasonal temperature [62], which may also have affected the microplastic intake fluctuations between seasons. In autumn, the metabolic rate was observed as low and the energy absorbed was equal to the energy demand, while, in spring, stress results in rapid decline of oxygen consumption [63]. In this regard, it has to be considered that, up to now, no harmonized criteria in terms of seasonal- or event-based timing of sampling have been defined. Environmental factors such as temperature affect filtration rates [64], and seasonal differences can also be influenced by events such as heavy rain and land runoff or higher river discharges [65,66,67,68].

*Mytilus edulis* is one of the most investigated species in terms of MP concentrations. Compared to findings on the French–Belgian–Dutch coastline (0.2 ± 0.3 items/g, lower size cutoff 5 μm, [39]), Norway (1.85 ± 3.74 items/g, lower size cutoff 15 mm, [15]; 0.97 items/g, lower size cutoff 70 μm, [19]), the United Kingdom (0.7 and 2.9 items/g, lower size cutoff 1 μm, [38]), and Denmark and the Netherlands Wadden Sea (0.32 items/g, lower size cutoff 5 μm, [69]), our results are slightly higher, with a median of 3.0 items/g. Compared to our study along the northwestern German coastline [24] and considering a common lower size cutoff of 63 µm, the results are in line, with 1.4 items/g (Schleswig-Holstein) to 1.2 items/g (Lower Saxony). Again, major differences can be related to different methodological approaches, such as digestion and density separation approaches and particle identification methods. Above all, the different lower size cutoffs, varying from 1 μm [39] to 150 μm [15], affected the results significantly.

*Mytilus edulis* was the only species showing a correlation between fibers and the individuals characteristics of net weight, length, and width. This raises the question of whether this filter feeder is more prone to uptake, due to a specific physical behavior, of fibers in suspension compared to fibers that are buried in sediments or adhered to the sediment surface layer. Based on our findings, this cannot be fully explained and should be further investigated within dedicated laboratory exposure tests. The same applies for the differences of fragment sizes according to sampling stations, which most likely relate to hydrodynamic conditions during tides. The polymer compositions determined according to a subset of particles are similar to those found for the other investigated species, consisting predominantly of PE (fragments) and PET (fibers).

Even considering different methodological approaches, a global comparison of MP abundance in biota showed that the results for both invertebrates and fish from the North Sea and Baltic Sea had significantly lower concentrations than, for example, in areas of the Mediterranean Sea and the Asian region [70,71,72,73,74].

MP concentrations detected in the digestive tract of *Platichthys flesus* also varied according to the sampling area (with the lowest concentrations in the area of Außenweser) but not according to the sampling campaign. Differences between the areas can be attributed to their location directly within the estuary of the rivers Ems (Borkum) and Weser (Außenweser) and in differences related to the distance to the shoreline, with the maximum distance represented by Baltrum, which is furthermore located behind the barrier isles of Norderney and Baltrum. Compared to other studies on *Platichthys flesus* in the North and Baltic Seas, the concentrations determined here were considerably higher. The results from other studies ranged on average from 0.05 [33] to 2.04 [71] particles/individual, compared to the 12 particles/individual determined here along the coast of Lower Saxony. The differences here were largely due to methodological differences. For example, Rummel et al. set a lower detection limit of 500 µm [32], which leaves out the majority of particles between 20 and 500 µm considered here. This also applies to studies that did not specify a lower detection limit, but which can be estimated to be >300 µm with manual selection of potential particles using tweezers [31,32]. In general, studies on MPs in fish species of the North and Baltic Seas largely found no differences between the respective stations studied, even as a function of nearshore and offshore regions [24,28,29,31,33]. A lack of significant differences between the sampled stations was also noted by Bråte et al. [22], who however identified a “hotspot” within the port of Bergen. Lenz et al. considered MP abundances in demersal and pelagic fish species (*Gadus morhua* and *Clupea harengus*), comparing habitats, marine areas, and coastal vs. offshore sites [28]. In this study, microplastics were found in 30% of all *Clupea harengus* individuals at coastal sites and in 16% of individuals at offshore sites, with a significantly higher exposure of individuals in the North Sea compared to the Baltic Sea. *Clupea harengus,* as a pelagic species, had significantly higher concentrations than the demersal species *Gadus morhua*. In addition, Foekema et al. concluded that for the seven species studied in their investigation, there were spatial differences, with higher levels in the southern North Sea compared to the northern North Sea with generally low concentrations [74]. Moreover, in a methodologically comparable study along the Schleswig-Holstein coastline of Germany, no spatial differences or gradients were found with respect to the concentrations occurring in fish [24]. A lack of spatial differences was also shown by [20] for *Clupea harengus* and *Sprattus sprattus* in the Baltic Sea. In addition, they did not detect significant temporal differences over an 18-year period (1987–2005) nor between the species studied or for sampling times [20]. Comparing demersal and pelagic species, it was demonstrated that demersal species, and in particular *Gadus morhua* (Atlantic cod), had higher abundances of MP than pelagic species [28,32,33,75]. However, other studies found no significant difference between these habitats [30]. Positive correlations were found between MP concentrations and individual size [20]. In this context, the differences that occurred between spring and summer sampling times were attributed to increased foraging, with seasonal increases in individual size. In contrast, a study of MP ingestion by *Gadus morhua* and *Pollachius virens* (coalfish) in Icelandic waters showed no relationship of MP concentrations with individual size or weight, and no relationship with gut filling, in contrast to [23].

In terms of particle characteristics, the differences in particle length were small; however, the significant differences between single stations cannot be explained here based on the location of the sampling regions, in terms of estuaries, barrier isles, or distance to the shoreline. It is assumed that both MP concentrations and particle characteristics in fish reflect the highly variable conditions in marine waters, which are less suitable for interlinking compared to the MP in invertebrates and the influencing factors, e.g., particle deposition. Similarly to the polymer composition of MP particles found in invertebrates, the MP in *Platichthys flesus* mainly consisted of PE (fragments) and PET (fibers).

### 4.2. MP Concentrations in Sediment Cores

MP concentrations determined within sediment cores from the invertebrate sampling stations did not show clear distribution patterns with depth, only at the station Cappel-Neufeld could a tendency of declining concentrations with increasing depth be identified. The mudflats of the Wadden Sea form a highly active layer that is, not only characterized by sediment relocations through tides [76], but is above all strongly affected by bioturbation processes. The feeding technique of species can affect the potential MP uptake, assuming that filter feeders are more prone to ingestion than deposit feeders [77]. Furthermore, the bioturbation activities of species vary, leading to particle diffusion or promoting the burial of MPs [78]. Due to this and the resulting large and varying range of values, and as only 12 sediment cores were retrieved, we did not assess a potential correlation between sediment and invertebrate MP concentrations. To assess the suitability of potential MP monitoring in mudflat sediments, further research especially addressing spatially high-resolution approaches in this highly active layer of sediment relocation is required.

To compare the results from sediment analyses, only the results from the 0–5 cm layer were taken into account, since in most studies only the top layer of sediment is considered. The MP concentrations ranged from 1813 (Neuharlingersiel 2020) to 5750 part/kg dry weight (Cappel-Neufeld 2020) and were considerably higher compared to a study from the Dutch coast assessing MP particle concentrations >10 µm, which determined mean values of 770 part/kg dry weight in a sediment sample of the Danziggat at the south of the isle of Ameland [38].

The polymer composition of MP particles within the sediment cores revealed a tendency of increasing MP particle abundance and higher material density with increasing depth. However, the number of analyzed particles was not representative enough to prove the significance of this statement. Strikingly, the polymer composition in sediments compared to those determined for biota differed, with increased amounts of high-density polymers such as PVC in sediments, which could not be detected for the biota samples. However, it would be expected that the composition in the deposit feeder Arenicola marina, in particular, would be similar to that of the sediment samples. Whether this bias is based on the potential selective feeding of Arenicola marina cannot be assessed here, due to the low particle numbers assessed for polymer composition, but indicates the necessity for further dedicated research in this regard.

### 4.3. Suitability of Investigated Species for MP-Biomonitoring

Regarding the suitability of the studied species for MP biomonitoring purposes, we identified several criteria as being met: (i) spatial representation for the German North Sea coast, Europe-wide, and globally; (ii) feasibility in terms of sampling and sample processing; and (ii) species-specific limitations regarding morphology and habitat mobility.

The habitat of *Arenicola marina* is widespread throughout Europe and extends over the northern Atlantic Ocean (also occurring along the east coast of America), arctic regions, the southern Baltic Sea, and regions of the Mediterranean [79,80]. Sampling is more time consuming than for other species but nevertheless very straightforward; within one tide, about 20 to 50 individuals can be collected. Further sample preparation via digestion in the laboratory proved to be easy, as the entire individual can be dissolved without prior dissection. To ensure the detection of concentrations with signals significantly above the detection limit, pooling of samples with three to five individuals is recommended. The determined concentrations per individual *Arenicola marina* were almost normally distributed, but taking into account the individual weights, they were clearly distributed in a right-skewed manner. For the development of a baseline and threshold values, a normal distribution in terms of comparability of sites should be aimed for. If necessary, this could be achieved by transforming the values when considering the reference unit particles per weight. With regard to species-specific characteristics, it should be taken into account that the individual sizes of *Arenicola marina* vary at different stations. A minimum size of 6–10 cm length at sampling and the pooling of individuals of similar length in two size classes are recommended. However, MP concentrations in *Arenicola marina* are highly dependent on the current gut filling status and, therefore, more or less reflect a sediment signal. Furthermore, we could identify a bias in terms of individual length. However, *Arenicola marina* represents a key indicator species in the specific ecosystem of the Wadden Sea, they should not be considered as a biota monitoring species for MP according to their time consuming sampling process and inconsistent data results, which possibly fluctuate with the gut filling status.

*Littorina littorea* is very well suited as an indicator species, due to its ubiquitous occurrence with good statistical representativeness of the value distributions and, in particular, due to the detection of potential synergies with other contaminants. A limitation is the low number of studies on this species under real environmental conditions, which indicates a low acceptance for monitoring programs. *Littorina littorea* is widely distributed globally and omnipresent along the German coast, with a lower occurrence along the Baltic sea coast [81]. *Littorina littorea* does not occur in the same abundance in every location selected or does not appear at all. Sampling is uncomplicated and quick. About 50–150 individuals can be collected in a very short time, depending on their abundance at the respective site. Laboratory processing is time-consuming and challenging, since the shell and muscle tissue need to be separated. During this process, it cannot be excluded that tissue residues adhering to the shell remain. Ideally, this preparation step should be further improved, which poses a challenge, since an initial treatment with hydrochloric acid to destroy the shell may lead to destruction of synthetic polymers and should therefore be refrained from. Mechanical disruption prior to digestion of the entire individuals, including shell fragments, and subsequent removal of the remaining shell debris could be considered. *Littorina littorea* shows overall concentrations per individual and per weight of an approximately normal distribution, which could be further improved by statistical transformation of the values. This suggests the suitability of *Littorina littorea* as an indicator species. Another potential option is using *Littorina littorea* as a species for evaluating near-surface accumulations from sedimentary or mineral surfaces, as they are predominantly grazers. However, it should be taken into account that the MP particles recorded here reflect the local characteristics of these surfaces. They result from the topographic position and the exposure in the terrain, although they can, for example, control the expression of algae populations. Accordingly, if *Littorina littorea* is considered for MP monitoring in biota, it is recommended that sampling should be conducted simultaneously to the monitoring of tributyltin (TBT) at the same locations and times. In addition to improving the comparability of stations further offshore, this would also result in synergies between the potential exposure to pollutants and MP.

In the context of MP monitoring, *Mytilus edulis* is a very good indicator candidate. Valid data are generated through pooled samples and the differentiation of dissimilar size classes that, in association with contaminant monitoring, provide valuable clues to the potentially harmful effects of MP. The inclusion of *Mytilus edulis* in biota monitoring has a high scientific acceptance and is already being considered for the development of such monitoring strategies [18]. *Mytilus edulis* is widely distributed along the German coast of the North Sea and Baltic Sea. Globally, the occurrence of this species is also nearly universal and includes the Baltic Sea, Northeast Atlantic, Mediterranean Sea, east and west coasts of North America, southern coastal sections of South America, southern coasts of Australia, and isolated occurrences along the coasts of Southeast Asia [82]. Sampling of *Mytilus edulis* is site-specific and simple when abundances are sufficient. This also applies to the sample preparation in the laboratory with easy and quick dissection of the soft tissue. The distribution of MP values in *Mytilus edulis* related to individuals and weight is approximately normal and can be further improved through suitable statistical transformation. *Mytilus edulis* is the only species studied in this investigation showing a significant difference in terms of individual characteristics for dimensions and weights. As a consequence, it is recommended that two different size classes should be considered as part of potential monitoring of MP in *Mytilus edulis*. The potential correlation between MP concentration and individual length or weight should also be recorded, particularly with respect to specifying valid and consistent reference units of MP load per individual vs. MP load per weight of tissue analyzed. Since the duration of the filtering phases of *bivalvia* (i.e., during tides) is a relevant factor, the respective topographic position above sea level and the related filter duration should also be documented. As a *bivalvia* and filter feeder, *Mytilus edulis* is in focus as a representative species and can therefore also reflect MP concentrations in waters. Similarly to *Littorina littorea*, future measurement campaigns using *Mytilus edulis* should seek a synergy with pollutant monitoring. Sampling of *Mytilus edulis* in synchronization with contaminant monitoring of mussel beds is strongly recommended.

Lastly, *Platichthys flesus* also turned out to be well suited for MP monitoring. Additionally, the inclusion of another pelagic fish species should be discussed. *Platichthys flesus* represents demersal fish and occurs universally and in high abundance along the German coasts of the North Sea and Baltic Sea. Relevant occurrences also exist along the east coast of North America [82]. Sampling and sample preparation are simple but require greater resources for vessel-based sampling campaigns. Furthermore, a very good synergy with the monitoring of contaminants in biota can be achieved. The integration of fish species in MP monitoring has a wide scientific acceptance; however, the current state of research more often includes pelagic species such as *Clupea harengus*.

## 5. Conclusions

MPs were present in all investigated invertebrate, fish, and sediment samples of the German North Sea coastline of Lower Saxony. Differences in MP concentrations and characteristics according to different sampling locations were most likely attributable to deposition and accumulation patterns driven by tides, currents, and exposure to potential MP sources within river estuaries.

Considering the results of the MP concentrations detected in terms of a spatial representation of the German North Sea coast, Europe wide, and globally; the feasibility in terms of sampling and sample processing; and species-specific limitations regarding morphology and habitat mobility, this study suggests *Mytilus edulis* as a filter feeder and *Platichthys flesus* as a demersal species for MP monitoring of biota. The inclusion of another pelagic species, such as *Clupea harengus,* should be contemplated. *Littorina littorea* may also be considered; however, further preliminary studies need to be conducted based on individuals collected during TBT effect monitoring. *Arenicola marina* is not considered suitable for MP monitoring in biota, due to the dependency on the current status of gut filling with sediment of individuals. The inclusion of sediment analyses from the upper sediment layers is recommended; however, it is not recommended to include depth profile sampling using sediment core sections, due to the highly active bioturbation processes in mudflats.

## Figures and Tables

**Table 1:** Characteristics of the sample locations.

Location	Geographical Exposition	Flow Exposure	Watt Type	Anthropogenic Pressure	River Basin District
Knockster Tief	west	eastern edge Ems estuary	mixed mudflats/mudflats	low	Ems (transitional waters)
Leybucht	north-west	bay location eastern edge Ems estuary	mixed mudflats/mudflats	low	Ems (polyhaline tidal flats)
Neuharlingersiel	north	main estuary Neuharlingersiel-low	mixed mudflats/mudflats	high harbor (fishing, boats, ferries, camping site, beach, residential area)	Ems (euhaline tidal flats)
Jadebusen	east	western edge Jade	mixed mudflats/mudflats	medium—high camping site, beach, residential area	Weser (euhaline tidal flats)
Tettens	east	western edge Weser estuary	mixed mudflats/mudflats	medium camping site, residential area	Weser (transitional waters)
Cappel- Neufeld	west	eastern edge Weser estuary	mixed mudflats/mudflats	low camping site	Weser (transitional waters)

**Table 2:** Total number and percentage of pooled species affected by microplastic contamination.

Species	*n* (Investigated/Affected)	Affected (%)
*Arenicola marina*	98/89	91
*Littorina littorea*	43/40	94
*Mytilus edulis*	142/121	85
*Platichthys flesus*	310/244	79

**Table 3:** Percentage of fragments, fibers, and microbeads per species (% of all particles).

Species	Fragment	Fiber	Microbead
*Arenicola marina*	92	5	3
*Littorina littorea*	91	5	4
*Mytilus edulis*	93	3	4
*Platichthys flesus*	92	6	2

## Data Availability

Not applicable.

## Supplementary Materials

The following supporting information can be downloaded at: https://www.mdpi.com/article/10.3390/ani13101698/s1, Table S1a: Descriptive statistics of MP particles (items/g) in biota. Table S1b: Descriptive statistics of MP fibers (items/g) in biota. Table S1c: Descriptive statistics of MP fragments (items/g) in biota; Table S2a: Descriptive statistics of MP particles (items/individual) in biota. Table S2b: Descriptive statistics of MP fibers (items/individual) in biota. Table S2c: Descriptive statistics of MP fragments (items/individual) in biota. Table S3: Amount of particles, fragments, fibers, and microbeads in sediment samples.
