# Supplementary material for: Abundance and Distribution of Microplastics in Invertebrate and Fish Species and Sediment Samples along the German Wadden Sea Coastline
Source: Animals (Basel). 2023 May 20;13(10):1698. doi: 10.3390/ani13101698 (PMC10215328; doi:10.3390/ani13101698)
Supplement: Supplementary file 1 [file animals-13-01698-s001.zip › animals-2385903-supplementary.pdf]

Table S1. (a) Descriptive statistics of MP particles (items/g) in biota.

Species	Feeding Type	Location	Year	Mean	Median	Min	Max	Stdev	Variance	Mean gross weight	Mean net weight	1st. qu.	3rd. qu.	N
Arenicola marina	deposit feeder	Knockster Tief	2019	18.43	4.28	0.74	79.74	28.92	836.34	17.38	-	2.67	30.72	21
			2020	10.16	10.04	0.00	22.66	8.87	78.64	7.69	-	1.96	18.42	13
		Leybucht	2019	59.69	40.84	11.10	248.08	57.83	3344.64	2.37	-	29.19	67.04	53
			2020	1.42	0.40	0.00	4.38	1.74	3.04	14.36	-	0.03	3.25	33
		Neuharlingersiel	2019	57.57	39.18	4.53	146.72	44.41	1972.69	2.40	-	31.18	83.08	39
			2020	0.46	0.43	0.41	0.56	0.06	0.00	24.47	-	0.41	0.51	25
		Jadebusen	2019	-	-	-	-	-	-	-	-	-	-	0
			2020	-	-	-	-	-	-	-	-	-	-	0
		Tettens	2019	16.13	3.05	0.00	72.95	24.28	589.36	3.18	-	0.00	31.72	32
			2020	3.61	3.86	0.00	7.50	3.13	9.81	10.80	-	0.21	6.86	24
		Cappel-Neufeld	2019	15.71	11.43	0.70	45.93	16.03	256.88	13.99	-	1.99	29.67	28
			2020	1.51	1.12	0.00	4.66	1.41	1.98	14.32	-	0.37	2.54	40
Littorina littorea	grazer	Knockster Tief	2019	2.77	2.78	0.77	4.75	1.51	2.29	24.16	5.85	1.51	4.00	61
			2020	1.95	1.77	1.77	2.31	0.31	0.10	15.52	3.93	1.77	0.00	45
		Leybucht	2019	5.72	4.92	1.60	11.59	3.95	15.58	47.78	11.90	2.31	9.52	68
			2020	2.24	2.40	0.00	4.32	2.16	4.68	34.92	7.76	0.00	0.00	45

		Neuharlingersiel	2019	9.09	5.37	5.01	17.49	5.99	35.91	20.63	6.16	5.14	16.46	80
			2020	1.89	0.97	0.47	4.23	2.04	4.17	68.93	3.96	0.47	0.00	36
		Jadebusen	2019	4.21	2.17	0.00	11.65	5.09	25.92	18.71	6.02	1.37	11.20	106
			2020	6.44	8.07	2.52	8.73	3.41	11.63	18.72	5.80	2.52	0.00	44
		Tettens	2019	-	-	-	-	-	-	-	-	-	-	0
			2020	-	-	-	-	-	-	-	-	-	-	0
		Cappel-Neufeld	2019	1.28	0.98	0.27	3.21	1.13	1.27	39.90	10.40	0.53	2.17	78
			2020	0.68	0.68	0.00	1.35	0.95	0.91	41.42	11.22	0,00	0.00	20
Mytilus edulis	filter feeder	Knockster Tief	2019	6.54	4.99	0.55	20.81	5.95	35.36	33.75	15.10	1.39	10.21	117
			2020	3.50	2.07	0.00	18.59	5.13	26.36	53.49	16.12	0.13	4.71	60
		Leybucht	2019	4.64	4.51	0.33	9.52	3.19	10.16	46.08	14.42	1.75	7.93	67
			2020	0.46	0.00	0.00	2.49	0.87	0.75	37.99	21.77	0.00	0.31	60
		Neuharlingersiel	2019	13.06	12.16	10.13	18.14	3.16	9.97	40.29	16.85	10.58	15.99	24
			2020	1.82	0.05	0.00	9.52	3.11	9.67	21.66	7.56	0.00	3.52	49
		Jadebusen	2019	4.39	4.02	0.91	9.33	2.32	5.39	22.99	8.16	2.67	4.61	113
			2020	0.20	0.23	0.00	0.52	0.21	0.04	21.22	6.94	0.00	0.37	33
		Tettens	2019	4.30	3.95	0.66	9.05	2.60	6.74	36.18	17.32	1.92	6.18	50
			2020	1.93	1.67	0.00	5.56	2.10	4.40	33.92	13.21	0.00	3.38	31
		Cappel-Neufeld	2019	8.59	4.12	1.08	33.86	10.42	108.61	37.67	16.23	1.60	16.79	53

			2020	0.81	0.45	0.00	3.31	1.19	1.41	53.93	21.16	0.00	1.11	37
Platichthys flesus	demersal	Borkum	2018	2.38	0.58	0.00	47.32	6.89	47.49	140.44	7.50	0.22	2.38	47
			2020	0.66	0.14	0.00	3.79	1.04	1.09	164.78	9.38	0.00	0.70	41
		Baltrum	2018	1.31	0.50	0.00	11.67	2.00	4.02	164.11	8.28	0.14	1.74	53
			2020	2.00	0.76	0.00	16.46	3.32	11.00	140.19	11.24	0.13	2.33	41
		Außenjade	2018	2.08	1.02	0.00	17.80	3.16	9.98	174.14	11.38	0.39	2.35	43
			2020	-	-	-	-	-	-	-	-	-	-	0
		Außenweser	2018	0.70	0.45	0.00	4.48	0.90	0.80	181.79	14.66	0.00	0.95	44
			2020	0.75	0.46	0.00	3.68	0.97	0.93	177.72	11.41	0.00	0.90	41

Table S1. (b) Descriptive statistics of MP fibers (items/g) in biota.

Species	Feeding Type	Location	Year	Mean	Median	Min	Max	Stdev	Variance	Mean gross weight	Mean net weight	1st. qu.	3rd. qu.	N
Arenicola marina	deposit feeder	Knockster Tief	2019	1.12	0.20	0.00	3.92	1.71	2.91	17.38	-	0.00	3.27	21
			2020	0.31	0.23	0.00	0.84	0.34	0.12	7.69	-	0.03	0.64	13
		Leybucht	2019	2.78	1.87	0.00	15.38	3.44	11.81	2.37	-	1.11	3.72	53
			2020	0.05	0.00	0.00	0.33	0.11	0.01	14.36	-	0.00	0.07	33
		Neuharlingersiel	2019	1.15	1.18	0.00	2.62	0.72	0.51	2.40	-	0.72	1.65	39
			2020	0.08	0.07	0.04	0.16	0.05	0.00	24.47	-	0.04	0.13	25

		Jadebusen	2019	-	-	-	-	-	-	-	-	-	-	0
			2020	-	-	-	-	-	-	-	-	-	-	0
		Tettens	2019	0.45	0.26	0.00	1.38	0.52	0.27	3.18	-	0.00	0.85	32
			2020	0.18	0.03	0.00	0.65	0.27	0.07	10.80	-	0.00	0.46	24
		Cappel-Neufeld	2019	0.24	0.00	0.00	1.07	0.41	0.17	13.99	-	0.00	0.49	28
			2020	0.13	0.07	0.00	0.49	0.15	0.02	14.32	-	0.00	0.22	40
Littorina littorea	grazer	Knockster Tief	2019	0.16	0.03	0.00	0.81	0.32	0.10	24.16	5.85	0.00	0.26	61
			2020	0.08	0.00	0.00	0.25	0.14	0.02	15.52	3.93	0.00	0.00	45
		Leybucht	2019	0.09	0.04	0.00	0.36	0.15	0.02	47.78	11.90	0.00	0.19	68
			2020	0.00	0.00	0.00	0.00	0.00	0.00	34.92	7.76	0.00	0.00	45
		Neuharlingersiel	2019	0.19	0.17	0.01	0.48	0.16	0.03	20.63	6.16	0.06	0.28	80
			2020	0.00	0.00	0.00	0.00	0.00	0.00	68.93	3.96	0.00	0.00	36
		Jadebusen	2019	0.04	0.02	0.00	0.16	0.06	0.00	18.71	6.02	0.00	0.04	106
			2020	0.20	0.14	0.00	0.46	0.24	0.06	18.72	5.80	0.00	0.00	44
		Tettens	2019	-	-	-	-	-	-	-	-	-	-	0
			2020	-	-	-	-	-	-	-	-	-	-	0
		Cappel-Neufeld	2019	0.03	0.00	0.00	0.09	0.05	0.00	39.90	10.40	0.00	0.08	78
			2020	0.02	0.02	0.00	0.03	0.02	0.00	41.42	11.22	0.00	0.00	20
Mytilus edulis		Knockster Tief	2019	0.34	0.21	0.02	0.97	0.32	0.10	33.75	15.10	0.06	0.66	117

	filter feeder		2020	0.06	0.06	0.00	0.17	0.07	0.00	53.49	16.12	0.00	0.12	60
		Leybucht	2019	0.14	0.12	0.00	0.46	0.11	0.01	46.08	14.42	0.07	0.14	67
			2020	0.03	0.00	0.00	0.39	0.11	0.01	37.99	21.77	0.00	0.00	60
		Neuharlingersiel	2019	0.08	0.00	0.00	0.23	0.11	0.01	40.29	16.85	0.00	0.20	24
			2020	0.04	0.00	0.00	0.25	0.08	0.01	21.66	7.56	0.00	0.02	49
		Jadebusen	2019	0.46	0.32	0.12	2.61	0.51	0.26	22.99	8.16	0.22	0.46	113
			2020	0.07	0.00	0.00	0.28	0.13	0.02	21.22	6.94	0.00	0.23	33
		Tettens	2019	0.12	0.09	0.00	0.54	0.17	0.03	36.18	17.32	0.00	0.19	50
			2020	0.06	0.05	0.00	0.17	0.07	0.01	33.92	13.21	0.00	0.11	31
		Cappel-Neufeld	2019	0.29	0.35	0.00	0.60	0.20	0.04	37.67	16.23	0.07	0.47	53
			2020	0.02	0.00	0.00	0.06	0.03	0.00	53.93	21.16	0.00	0.50	37
Platichthys flesus	demersal	Borkum	2018	0.08	0.00	0.00	1.05	0.19	0.04	140.44	7.50	0.00	0.10	47
			2020	0.02	0.00	0.00	0.33	0.07	0.00	164.78	9.38	0.00	0.00	41
		Baltrum	2018	0.06	0.00	0.00	0.57	0.11	0.01	164.11	8.28	0.00	0.08	53
			2020	0.01	0.00	0.00	0.16	0.04	0.00	140.19	11.24	0.00	0.00	41
		Außenjade	2018	0.05	0.00	0.00	0.51	0.10	0.01	174.14	11.38	0.00	0.08	43
			2020	-	-	-	-	-	-	-	-	-	-	0
		Außenweser	2018	0.02	0.00	0.00	0.29	0.06	0.00	181.79	14.66	0.00	0.00	44
			2020	0.02	0.00	0.00	0.28	0.05	0.00	177.72	11.41	0.00	0.00	41

Table S1. (c) Descriptive statistics of MP fragments (items/g) in biota.

Species	Feeding Type	Location	Year	Mean	Median	Min	Max	Stdev	Variance	Mean gross weight	Mean net weight	1st. qu.	3rd. qu.	N
Arenicola marina	deposit feeder	Knockster Tief	2019	8.66	1.59	0.00	73.20	19.92	396.62	17.38	-	0.00	4.86	21
			2020	4.93	0.24	0.00	22.22	7.73	59.77	7.69	-	0.00	10.08	13
		Leybucht	2019	28.45	6.18	0.00	230.77	47.61	2266.34	2.37	-	0.00	39.31	53
			2020	0.68	0.00	0.00	4.38	1.36	1.86	14.36	-	0.00	0.55	33
		Neuharlingersiel	2019	28.21	6.70	0.00	136.89	39.86	1589.00	2.40	-	0.47	37.97	39
			2020	0.19	0.16	0.00	0.42	0.20	0.04	24.47	-	0.00	0.38	25
		Jadebusen	2019	-	-	-	-	-	-	-	-	-	-	0
			2020	-	-	-	-	-	-	-	-	-	-	0
		Tettens	2019	7.84	0.00	0.00	72.95	18.53	343.41	3.18	-	0.00	2.47	32
			2020	1.72	0.20	0.00	6.98	2.60	6.78	10.80	-	0.00	3.64	24
		Cappel-Neufeld	2019	7.74	1.36	0.00	43.65	12.98	168.59	13.99	-	0.70	11.39	28
			2020	0.69	0.07	0.00	4.52	1.16	1.35	14.32	-	0.00	1.09	40
Littorina littorea	grazer	Knockster Tief	2019	1.13	0.39	0.00	3.76	1.48	2.20	24.16	5.85	0.00	2.57	61
			2020	0.93	0.76	0.00	2.31	1.05	1.11	15.52	3.93	0.00	1.90	45
		Leybucht	2019	2.71	0.80	0.00	11.09	3.75	14.08	47.78	11.90	0.05	5.04	68

			2020	1.12	0.00	0.00	4.32	1.84	3.38	34.92	7.76	0.00	2.88	45
		Neuharlingersiel	2019	4.16	3.23	0.08	14.75	4.67	21.81	20.63	6.16	0.20	5.10	80
			2020	0.95	0.32	0.00	4.23	1.64	2.69	68.93	3.96	0.00	1.66	36
		Jadebusen	2019	1.99	0.48	0.00	11.13	3.93	15.46	18.71	6.02	0.00	2.32	106
			2020	3.12	1.17	0.00	8.59	3.91	15.25	18.72	5.80	0.17	7.81	44
		Tettens	2019	-	-	-	-	-	-	-	-	-	-	0
			2020	-	-	-	-	-	-	-	-	-	-	0
		Cappel-Neufeld	2019	0.62	0.18	0.00	3.12	0.96	0.92	39.90	10.40	0.00	0.91	78
			2020	0.30	0.03	0.00	1.29	0.67	0.45	41.42	11.22	0.00	0.98	20
Mytilus edulis	filter feeder	Knockster Tief	2019	3.10	0.60	0.00	20.14	5.06	25.62	33.75	15.10	0.00	4.46	117
			2020	1.72	0.12	0.00	18.39	3.89	15.13	53.49	16.12	0.00	2.10	60
		Leybucht	2019	2.25	0.34	0.00	8.95	3.09	9.55	46.08	14.42	0.00	4.57	67
			2020	0.21	0.00	0.00	2.09	0.59	0.34	37.99	21.77	0.00	0.00	60
		Neuharlingersiel	2019	6.49	5.38	0.42	17.09	6.37	40.58	40.29	16.85	0.75	11.56	24
			2020	0.89	0.00	0.00	9.52	2.34	5.46	21.66	7.56	0.00	0.00	49
		Jadebusen	2019	1.96	0.87	0.00	8.59	2.41	5.79	22.99	8.16	0.00	3.58	113
			2020	0.06	0.00	0.00	0.52	0.16	0.03	21.22	6.94	0.00	0.00	33
		Tettens	2019	2.09	0.87	0.00	8.01	2.55	6.48	36.18	17.32	0.05	4.05	50
			2020	0.93	0.03	0.00	5.01	1.57	2.46	33.92	13.21	0.00	1.82	31

		Cappel-Neufeld	2019	4.15	1.38	0.00	31.66	7.68	59.02	37.67	16.23	0.02	4.00	53
			2020	0.40	0.00	0.91	0.82	0.00	3.31	53.93	21.16	0.00	0.49	37
Platichthys flesus	demersal	Borkum	2018	1.15	0.00	0.00	45.22	4.77	22.78	140.44	7.50	0.00	0.66	47
			2020	0.32	0.00	0.00	3.79	0.80	0.63	164.78	9.38	0.00	0.11	41
		Baltrum	2018	0.63	0.00	0.00	11.67	1.52	2.30	164.11	8.28	0.00	0.46	53
			2020	1.00	0.00	0.00	16.03	2.49	6.20	140.19	11.24	0.00	0.73	41
		Außenjade	2018	1.01	0.08	0.00	17.80	2.42	5.87	174.14	11.38	0.00	1.03	43
			2020	-	-	-	-	-	-	-	-	-	-	0
		Außenweser	2018	0.34	0.00	0.00	4.15	0.69	0.48	181.79	14.66	0.00	0.36	44
			2020	0.37	0.00	0.00	3.54	0.72	0.52	177.72	11.41	0.00	0.43	41

Table S2. (a) Descriptive statistics of MP particles (items/individual) in biota.

Species	Feeding Type	Location	Year	Mean	Median	Min	Max	Stdev	Variance	Mean gross weight	Mean net weight	1st. qu.	3rd. qu.	N
Arenicola marina	deposit feeder	Knockster Tief	2019	26.67	17.33	4.33	82.00	27.20	739.64	17.38	-	5.67	40.67	21
			2020	20.33	20.67	0.00	52.00	19.80	392.22	7.69	-	4.00	36.50	13
		Leybucht	2019	26.00	23.34	15.54	241.50	7.67	64.50	2.37	-	12.50	33.33	53
			2020	4.13	1.00	0.00	15.80	5.36	28.68	14.36	-	0.16	7.06	33

		Neuharlingersiel	2019	36.33	31.33	8.33	69.00	22.59	510.43	2.40	-	16.33	61.50	39
			2020	2.15	2.00	1.40	3.60	0.86	0.75	24.47	-	1.50	2.88	25
		Jadebusen	2019	-	-	-	-	-	-	-	-	-	-	0
			2020	-	-	-	-	-	-	-	-	-	-	0
		Tettens	2019	10.42	7.67	0.00	50.33	15.05	226.44	3.18	-	7.67	15.33	32
			2020	10.67	8.84	0.00	27.00	10.08	101.60	10.80	-	1.33	20.83	24
		Cappel-Neufeld	2019	25.15	18.67	6.00	94.00	27.61	762.37	13.99	-	8.16	30.33	28
			2020	6.35	4.50	0.00	21.00	6.27	39.37	14.32	-	1.16	10.00	40
Littorina littorea	grazer	Knockster Tief	2019	1.76	1.29	0.67	3.86	1.23	1.50	24.16	5.85	0.81	2.89	61
			2020	0.51	0.47	0.47	0.60	0.08	0.01	15.52	3.93	0.47	0.00	45
		Leybucht	2019	5.16	5.00	1.14	10.80	3.73	13.90	47.78	11.90	1.88	8.52	68
			2020	1.13	1.33	0.00	2.07	1.05	1.10	34.92	7.76	0.00	0.00	45
		Neuharlingersiel	2019	3.51	2.94	2.07	6.40	1.72	2.95	20.63	6.16	2.16	4.99	80
			2020	0.37	0.43	0.25	0.43	0.10	0.01	68.93	3.96	0.25	0.00	36
		Jadebusen	2019	1.65	0.87	0.00	4.60	2.03	4.12	18.71	6.02	0.56	4.47	106
			2020	6.12	3.20	2.75	12.40	5.45	29.66	18.72	5.80	2.75	0.00	44
		Tettens	2019	-	-	-	-	-	-	-	-	-	-	0
			2020	-	-	-	-	-	-	-	-	-	-	0
		Cappel-Neufeld	2019	0.90	0.80	0.20	2.33	0.86	0.73	39.90	10.40	0.24	1.60	78

			2020	0.74	0.74	0.00	1.47	1.04	1.08	41.42	11.22	0.00	0.00	20
Mytilus edulis	filter feeder	Knockster Tief	2019	15.83	9.40	1.40	57.25	14.96	223.78	33.75	15.10	5.15	23.45	117
			2020	11.03	8.40	0.00	56.00	15.51	240.48	53.49	16.12	0.40	14.40	60
		Leybucht	2019	13.70	12.90	1.20	37.25	10.61	112.60	46.08	14.42	5.26	21.10	67
			2020	1.15	0.00	0.00	4.00	1.57	2.47	37.99	21.77	0.00	2.40	60
		Neuharlingersiel	2019	45.81	42.00	36.25	62.20	10.59	112.18	40.29	16.85	37.32	56.20	24
			2020	3.06	0.09	0.00	16.00	5.23	27.35	21.66	7.56	0.00	5.95	49
		Jadebusen	2019	6.60	5.60	1.80	13.60	3.48	12.12	22.99	8.16	3.80	9.00	113
			2020	0.31	0.40	0.00	0.80	0.32	0.10	21.22	6.94	0.00	0.50	33
		Tettens	2019	13.98	10.10	2.40	31.80	10.01	100.24	36.18	17.32	6.65	21.70	50
			2020	5.68	5.44	0.00	12.20	5.26	27.70	33.92	13.21	0.00	11.30	31
		Cappel-Neufeld	2019	16.53	10.60	3.60	54.00	13.99	195.81	37.67	16.23	8.50	18.60	53
			2020	3.53	1.14	0.00	14.60	5.28	27.92	53.93	21.16	0.00	4.60	37
Platichthys flesus	demersal	Borkum	2018	9.06	6.00	0.00	45.00	10.40	108.19	140.44	7.50	2.00	12.00	47
			2020	4.63	1.00	0.00	24.00	6.81	46.34	164.78	9.38	0.00	6.00	41
		Baltrum	2018	8.43	4.00	0.00	44.00	10.03	100.63	164.11	8.28	1.00	11.50	53
			2020	18.07	8.00	0.00	156.00	28.49	811.67	140.19	11.24	1.00	24.00	41
		Außenjade	2018	16.02	11.00	0.00	89.00	18.11	328.12	174.14	11.38	5.00	18.00	43

			2020	-	-	-	-	-	-	-	-	-	-	0
		Außenweser	2018	10.73	5.50	0.00	55.00	12.92	166.90	181.79	14.66	0.00	17.75	44
			2020	9.56	6.00	0.00	61.00	13.26	175.95	177.72	11.41	0.00	11.00	41

Table S2. (b) Descriptive statistics of MP fibers (items/individual) in biota.

Species	Feeding Type	Location	Year	Mean	Median	Min	Max	Stdev	Variance	Mean gross weight	Mean net weight	1st. qu.	3rd. qu.	N
Arenicola marina	deposit feeder	Knockster Tief	2019	1.24	1.00	0.00	3.33	1.20	1.43	17.38	-	0.00	2.00	21
			2020	0.47	0.33	0.00	1.00	0.38	0.15	7.69	-	0.16	0.83	13
		Leybucht	2019	1.23	1.00	0.97	0.94	0.00	4.00	2.37	-	0.62	1.67	53
			2020	0.24	0.00	0.00	1.40	0.46	0.22	14.36	-	0.00	0.36	33
		Neuharlingersiel	2019	0.90	0.67	0.00	2.67	0.76	0.58	2.40	-	0.33	1.33	39
			2020	0.37	0.40	0.17	0.60	0.19	0.03	24.47	-	0.18	0.55	25
		Jadebusen	2019	-	-	-	-	-	-	-	-	-	-	0
			2020	-	-	-	-	-	-	-	-	-	-	0
		Tettens	2019	0.51	0.33	0.00	3.33	0.97	0.94	0.51	-	0.00	0.67	32
			2020	0.50	0.17	0.00	2.33	0.82	0.67	0.50	-	0.00	0.83	24
		Cappel-Neufeld	2019	0.59	0.00	0.00	2.33	0.88	0.77	0.59	-	0.00	1.33	28
			2020	0.45	0.33	0.00	1.33	0.46	0.21	0.45	-	0.00	0.87	40

Littorina littorea	grazer	Knockster Tief	2019	0.21	0.14	0.00	0.57	0.24	0.06	0.21	5.85	0.00	0.43	61
			2020	0.02	0.00	0.00	0.07	0.04	0.00	0.02	3.93	0.00	0.00	45
		Leybucht	2019	0.27	0.33	0.00	0.53	0.26	0.07	0.27	11.90	0.00	0.51	68
			2020	0.00	0.00	0.00	0.00	0.00	0.00	0.00	7.76	0.00	0.00	45
		Neuharlingersiel	2019	0.24	0.14	0.07	0.60	0.22	0.05	0.24	6.16	0.07	0.45	80
			2020	0.00	0.00	0.00	0.00	0.00	0.00	0.00	3.96	0.00	0.00	36
		Jadebusen	2019	0.10	0.07	0.00	0.27	0.11	0.01	0.10	6.02	0.00	0.20	106
			2020	0.23	0.20	0.00	0.50	0.25	0.06	0.23	5.80	0.00	0.00	44
		Tettens	2019	-	-	-	-	-	-	-	-	-	-	0
			2020	-	-	-	-	-	-	-	-	-	-	0
		Cappel-Neufeld	2019	0.03	0.00	0.00	0.07	0.04	0.00	39.90	10.40	0.00	0.07	78
			2020	0.10	0.10	0.00	0.20	0.14	0.02	41.42	11.22	0.00	0.00	20
Mytilus edulis	filter feeder	Knockster Tief	2019	0.68	0.55	0.20	2.00	0.52	0.27	33.75	15.10	0.28	0,95	117
			2020	0.20	0.20	0.00	0.60	0.21	0.04	53.49	16.12	0.00	0.40	60
		Leybucht	2019	0.43	0.40	0.00	1.40	0.35	0.13	46.08	14.42	0.20	0.56	67
			2020	0.05	0.00	0.00	0.60	0.17	0.03	37.99	21.77	0.00	0.00	60
		Neuharlingersiel	2019	0.28	0.00	0.00	0.80	0.39	0.15	40.29	16.85	0.00	0.70	24
			2020	0.06	0.00	0.00	0.40	0.13	0.02	21.66	7.56	0.00	0.04	49
		Jadebusen	2019	0.60	0.60	0.20	1.60	0.34	0.12	22.99	8.16	0.40	0.80	113

			2020	0.13	0.00	0.00	0.50	0.22	0.05	21.22	6.94	0.00	0.40	33
		Tettens	2019	0.34	0.40	0.00	1.00	0.39	0.15	36.18	17.32	0.00	0.60	50
			2020	0.18	0.10	0.00	0.50	0.22	0.05	33.92	13.21	0.00	0.42	31
		Cappel-Neufeld	2019	0.63	0.60	0.00	1.40	0.39	0.15	37.67	16.23	0.40	0.83	53
			2020	0.05	0.00	0.00	0.20	0.08	0.01	53.93	21.16	0.00	0.14	37
Platichthys flesus	demersal	Borkum	2018	0.40	0.00	0.00	3.00	0.74	0.55	140.44	7.50	0.00	1.00	47
			2020	0.12	0.00	0.00	3.00	0.51	0.26	164.78	9.38	0.00	0.00	41
		Baltrum	2018	0.39	0.00	0.00	2.00	0.63	0.39	164.11	8.28	0.00	1.00	53
			2020	0.40	0.00	0.00	2.00	0.63	0.40	140.19	11.24	0.00	0.00	41
		Außenjade	2018	0.51	0.00	0.00	5.00	0.96	0.92	174.14	11.38	0.00	1.00	43
			2020	-	-	-	-	-	-	-	-	-	-	0
		Außenweser	2018	0.34	0.00	0.00	4.00	0.78	0.60	181.79	14.66	0.00	0.00	44
			2020	0.24	0.00	0.00	5.00	0.83	0.69	177.72	11.41	0.00	0.00	41

Table S2. (c) Descriptive statistics of MP fragments (items/individual) in biota.

Species	Feeding Type	Location	Year	Mean	Median	Min	Max	Stdev	Variance	Mean gross weight	Mean net weight	1st. qu.	3rd. qu.	N
Arenicola marina		Knockster Tief	2019	12.71	2.33	0.00	78.33	21.81	475.86	17.38	-	0.00	17.50	21

	deposit feeder		2020	9.93	0.33	0.00	51.00	16.64	276.76	7.69	-	0.00	20.16	13
		Leybucht	2019	12.38	4.50	0.00	60.00	16.01	256.46	2.37	-	0.00	22.08	53
			2020	1.95	0.00	0.00	14.40	3.91	15.28	14.36	-	0.00	1.80	33
		Neuharlingersiel	2019	17.72	5.17	0.00	66.67	22.60	510.80	2.40	-	0.50	31.41	39
			2020	0.89	0.50	0.00	3.00	1.07	1.14	24.47	-	0.00	1.62	25
		Jadebusen	2019	-	-	-	-	-	-	-	-	-	-	0
			2020	-	-	-	-	-	-	-	-	-	-	0
		Tettens	2019	4.95	0.00	0.00	50.33	11.57	133.89	0.51	-	0.00	5.83	32
			2020	5.08	0.67	0.00	24.00	8.09	65.41	0.50	-	0.00	8.66	24
		Cappel-Neufeld	2019	12.28	4.50	0.00	89.33	21.58	465.87	0.59	-	0.33	18.17	28
			2020	2.95	0.29	0.00	20.33	5.13	26.35	0.45	-	0.00	4.06	40
Littorina littorea	grazer	Knockster Tief	2019	0.77	0.20	0.00	3.86	1.17	1.38	0.21	5.85	0.00	1.20	61
			2020	0.25	0.20	0.00	0.60	0.28	0.08	0.02	3.93	0.00	0.50	45
		Leybucht	2019	2.44	0.57	0.00	10.33	3.46	11.98	0.27	11.90	0.04	4.82	68
			2020	0.57	0.00	0.00	2.07	0.91	0.83	0.00	7.76	0.00	1.51	45
		Neuharlingersiel	2019	1.64	1.20	0.07	5.40	1.79	3.19	0.24	6.16	0.08	2.41	80
			2020	0.19	0.16	0.00	0.43	0.19	0.04	0.00	3.96	0.00	0.37	36
		Jadebusen	2019	0.78	0.20	0.00	4.33	1.56	2.44	0.10	6.02	0.00	0.90	106
			2020	2.94	1.13	0.00	12.20	4.69	22.00	0.23	5.80	0.15	5.30	44

		Tettens	2019	-	-	-	-	-	-	-	-	-	-	0
			2020	-	-	-	-	-	-	-	-	-	-	0
		Cappel-Neufeld	2019	0.43	0.13	0.00	2.27	0.70	0.49	39.90	10.40	0.00	0.73	78
			2020	0.32	0.04	0.00	1.40	0.73	0.53	41.42	11.22	0.00	1.06	20
Mytilus edulis	filter feeder	Knockster Tief	2019	7.58	2.10	0.00	54.50	12.42	154.29	33.75	15.10	0.00	8.20	117
			2020	5.42	0.40	0.00	55.40	11.86	140.58	53.49	16.12	0.00	8.75	60
		Leybucht	2019	6.64	1.28	0.00	35.00	9.62	92.61	46.08	14.42	0.00	13.30	67
			2020	0.55	0.00	0.00	4.00	1.17	1.37	37.99	21.77	0.00	0.00	60
		Neuharlingersiel	2019	22.77	19.18	1.50	58.60	22.25	495.17	40.29	16.85	2.70	40.50	24
			2020	1.50	0.00	0.00	16.00	3.93	15.44	21.66	7.56	0.00	0.00	49
		Jadebusen	2019	3.00	1.00	0.00	12.20	3.72	13.81	22.99	8.16	0.00	5.25	113
			2020	0.09	0.00	0.00	0.80	0.24	0.06	21.22	6.94	0.00	0.00	33
		Tettens	2019	6.82	3.00	0.00	29.80	8.95	80.12	36.18	17.32	0.25	9.15	50
			2020	2.75	0.10	0.00	11.00	4.27	18.24	33.92	13.21	0.00	5.57	31
		Cappel-Neufeld	2019	7.95	2.98	0.00	50.50	11.74	137.75	37.67	16.23	0.15	11.65	53
			2020	1.74	0.00	0.00	14.60	4.02	16.19	53.93	21.16	0.00	1.80	37
Platichthys flesus	demersal	Borkum	2018	4.33	0.00	0.00	43.00	8.26	68.16	140.44	7.50	0.00	5.25	47
			2020	2.26	0.00	0.00	24.00	5.22	27.21	164.78	9.38	0.00	1.00	41
		Baltrum	2018	4.02	0.00	0.00	42.00	7.88	62.02	164.11	8.28	0.00	4.00	53

			2020	8.98	0.00	0.00	152.00	21.44	459.68	140.19	11.24	0.00	8.00	41
		Außenjade	2018	7.76	1.00	0.00	88.00	14.44	208.47	174.14	11.38	0.00	11.00	43
			2020	-	-	-	-	-	-	-	-	-	-	0
		Außenweser	2018	5.19	0.00	0.00	51.00	10.18	103.72	181.79	14.66	0.00	5.00	44
			2020	4.66	0.00	0.00	52.00	9.60	92.23	177.72	11.41	0.00	6.00	41

Table S3. Amount of particles, fragments, fibers and microbeads in sediment samples.

Location	Year	Depth	Particles per kg DW	Fragments per kg DW	Fibers per kg DW	Microbeads per kg DW
Knockster Tief	2019	0-5	3,268	2,733	493	41
		5-10	2,635	2,210	405	20
		10-15	1,458	1,349	87	22
		15-20	3,793	2,919	762	112
	2020	0-5	2,424	2,255	94	75
		5-10	2,392	2,283	91	18
		10-15	1,637	1,425	106	106
		15-20	3,437	3,237	153	46
		20-25	1,620	1,451	135	34
Leybucht	2019	0-5	2,523	1,949	544	30
		5-10	2,719	2,056	578	85
		10-15	3,592	3,024	458	110
		15-20	3,177	2,854	247	76
		20-25	5,632	4,756	647	228
	2020	0-5	3,468	2,843	406	219
		5-10	3,226	2,517	284	425
		10-15	2,830	2,523	243	65
		15-20	2,083	1,697	370	17
		20-25	2,122	1,833	145	145
		25-30	4,344	3,942	302	101
Neuharlingersiel	2019	0-5	2,387	2,119	134	134
		5-10	1,710	1,640	71	0
		10-15	2,076	1,627	411	37
		15-20	5,150	4,419	365	365
		20-25	4,383	4,000	213	170
	2020	0-5	1,813	1,656	94	63
		5-10	2,832	2,406	305	122
		10-15	1,822	1,372	379	71
		15-20	2,401	2,173	143	86

Jadebusen	2019	0-5	5,456	4,430	349	677
		5-10	1,938	1,588	117	234
		10-15	4,160	3,499	220	440
		15-20	7,392	5,959	493	941
		20-25	2,908	2,529	190	190
	2020	0-5	4,842	4,447	264	132
		5-10	8,128	7,155	661	311
		10-15	4,557	4,210	217	130
Tettens	2019	0-5	4,620	4,018	563	39
		5-10	3,743	2,894	848	0
		10-15	3,462	3,017	444	0
		15-20	3,851	3,667	183	0
		20-25	4,619	4,077	541	0
		25-30	4,069	3,732	337	0
	2020	0-5	2,889	2,756	133	0
		5-10	1,232	1,211	21	0
		10-15	2,379	2,311	68	0
Cappel-Neufeld	2019	0-5	2,103	1,840	234	29
		5-10	747	685	62	0
		10-15	242	242	0	0
		15-20	132	132	0	0
		20-25	0	0	0	0
		25-30	0	0	0	0
	2020	0-5	5,750	4,443	105	1,202
		5-10	503	406	58	39
		10-15	55	55	0	0
		15-20	41	20	20	0
